# Simultaneous Normalization and Compensatory Changes in Right Hemisphere Connectivity during Aphasia Therapy

**DOI:** 10.3390/brainsci11101330

**Published:** 2021-10-08

**Authors:** Tammar Truzman, Elizabeth Rochon, Jed Meltzer, Carol Leonard, Tali Bitan

**Affiliations:** 1 Communication Sciences and Disorders Department and IIPDM, University of Haifa, Haifa 3498838, Israel; 2 The Integrated Brain and Behavior Research Center, University of Haifa, Haifa 3498838, Israel; 3 Department of Speech Language Pathology and Rehabilitation Sciences Institute, University of Toronto, Toronto, ON M5G 1V7, Canada; elizabeth.rochon@utoronto.ca (E.R.); jmeltzer@research.baycrest.org (J.M.); Carol.Leonard@uottawa.ca (C.L.); tbitan@research.haifa.ac.il (T.B.); 4 KITE Research Institute, Toronto Rehab, University Health Network (UHN), Toronto, ON M5G 2A2, Canada; 5 Psychology Department, University of Toronto, Toronto, ON M5S 1A1, Canada; 6 Rotman Research Institute, Baycrest Health Sciences, Toronto, ON M6A 2E1, Canada; 7 School of Rehabilitation Sciences, University of Ottawa, Ottawa, ON K1H 8L1, Canada; 8 Psychology Department and IIPDM, University of Haifa, Haifa 3498838, Israel

**Keywords:** effective connectivity, language network, DCM, PEB, aphasia, stroke, speech therapy, language recovery

## Abstract

Changes in brain connectivity during language therapy were examined among participants with aphasia (PWA), aiming to shed light on neural reorganization in the language network. Four PWA with anomia following left hemisphere stroke and eight healthy controls (HC) participated in the study. Two fMRI scans were administered to all participants with a 3.5-month interval. The fMRI scans included phonological and semantic tasks, each consisting of linguistic and perceptual matching conditions. Between the two fMRI scans, PWA underwent Phonological Components Analysis treatment. Changes in effective connectivity during the treatment were examined within right hemisphere (RH) architecture. The results illustrate that following the treatment, the averaged connectivity of PWA across all perceptual and linguistic conditions in both tasks increased resemblance to HC, reflecting the normalization of neural processes associated with silent object name retrieval. In contrast, connections that were specifically enhanced by the phonological condition in PWA decreased in their resemblance to HC, reflecting emerging compensatory reorganization in RH connectivity to support phonological processing. These findings suggest that both normalization and compensation play a role in neural language reorganization at the chronic stage, occurring simultaneously in the same brain.

## 1. Introduction

This study examines the effects of language therapy on brain functional connectivity among left hemisphere stroke patients with aphasia (PWA). Specifically, we examined whether phonological treatment affects brain connectivity in the intact right hemisphere, which was measured during phonological and semantic tasks, using pre- and post-treatment fMRI scans. We asked whether changes related to phonological treatment are limited to the processing of phonological aspects of language or whether they generalize to semantic processing. Moreover, we tested which treatment-related changes are associated with the normalization of brain connectivity and which are associated with emergence of compensatory connections not evident in the healthy brain. Finally, we asked whether normalization and compensatory changes differ for semantic and phonological tasks, which are known to engage distinct neural areas within the language network. 

Aphasia is an acquired language impairment typically following left hemisphere damage to frontal, parietal, and temporal brain regions [1]. Anomia, a persistent word-finding difficulty, is the most common symptom of aphasia [2]. In the last few decades, a great deal of knowledge has accumulated regarding aphasia profiles and treatment approaches [3]. This knowledge base has been further enhanced by the emergence of brain imaging and electrophysiological techniques. These techniques enable us to track the neural correlates of language rehabilitation. While language recovery in the subacute phase after stroke is often related to the restoration of temporary dysfunctional perilesional regions, longer-term recovery is thought to reflect neuroplasticity through functional reorganization [4]. Studies in this field are engaged in an ongoing discussion about the nature of the neural changes underlying recovery from left hemisphere damage [5,6,7,8]. Some studies emphasize the role of normalization of brain activation in language recovery, typically reflected in reactivation of perilesional regions in the left hemisphere [5,9,10,11,12,13]. Holding this view, some studies suggest the left hemisphere damage leads to collateral and transcallosal disinhibition, resulting in inefficient increased activation of the right hemisphere during language processing [14]. This hypothesis is supported by findings from brain stimulation studies, wherein the suppression of specific regions in the right hemisphere in non-fluent aphasia resulted in improved language performances [15,16,17,18]. In contrast, other studies suggest that the increased involvement of the right hemisphere (RH) in language processing following left hemisphere damage reflects compensatory supportive processes [8,19,20,21,22,23,24,25]. Recent work by Kiran et al. [26] suggests that although spared tissue within the left hemisphere is critically engaged in language recovery, right hemisphere regions are also involved in this process. 

Several factors may determine the role of normalization versus compensatory processes in language recovery. Saur et al. [27] suggested that these processes are dynamic, wherein compensation by the right hemisphere is upregulated in the acute phase, which is followed by normalization of the left hemisphere perilesional activity in the chronic stage. Others suggested that in the chronic phase of aphasia, the right hemisphere may contribute to language recovery but not always efficiently, and homotopic regions are not necessarily homologous in their function [7,8]. Furthermore, some studies argue that normalization and compensation depend on the nature of the tasks used for measuring language processing following recovery [28,29]. This implies that the dichotomous view of compensation versus normalization may be oversimplified, and that both processes may occur simultaneously. 

There is ample evidence from healthy adults that semantic processing in different modalities is bilaterally represented [30,31,32], whereas phonological processing is more left lateralized [33,34,35]. This notion is also suggested by the dual stream model of speech perception and its more recent extensions [36,37,38,39,40]. According to these models, the ventral stream, which extends from the posterior superior and middle temporal lobe to the anterior middle and inferior temporal lobe and to the ventral inferior frontal gyrus (IFG), connects auditory/phonological networks with conceptual semantic systems through hierarchical lexical–syntactic processing, for speech comprehension and production, and it is largely bilaterally organized. In contrast, the dorsal stream extends into the dorsal aspects of the left inferior frontal gyrus, including premotor areas. This route maps acoustic speech signals to frontal lobe articulatory networks and is strongly left lateralized. 

These two models are consistent with evidence for the distinct function of sub-portions of inferior frontal gyrus (IFG) within the left hemisphere. The posterior–dorsal pars opercularis is preferentially engaged in sublexical, phonologically-related processing, whereas the anterior–ventral pars triangularis contributes to lexico-semantic access and retrieval [41,42]. The hemispheric differences between phonological and semantic processing in healthy individuals can inform our expectations for treatment-related changes associated with normalization and compensation in PWA. Specifically, following LH stroke, changes in the intact RH during semantic tasks may reflect normalization due to the bilateral nature of semantic processing in healthy individuals. In contrast, post-treatment involvement of the RH in phonological processing may reflect compensatory changes due to the left lateralization of such processing before the stroke. Similarly, connectivity changes within sub-portions of IFG are expected to be associated with the relevant task. 

It is widely accepted that analysis of brain connectivity is of significant importance to the understanding of brain plasticity [3,43,44]. Changes in functional connectivity among PWA have been reported in several studies [45,46,47,48], and specific changes in the language network have been detected following language therapy [21,49,50]. Effective connectivity, which refers to the causal influence that neural populations exert on each other and can be inferred from fMRI data, has been analyzed in several language studies in healthy individuals [42,51,52,53,54,55,56,57]. Nevertheless, only a few studies have used this analysis to understand the network reorganization processes associated with language recovery in patients with aphasia [58,59,60,61,62,63,64]. Effective connectivity can provide crucial information regarding aphasia recovery profiles, as it captures causal changes at the network level, which is most relevant for reorganization processes. 

In the current study, effective connectivity changes in PWA were measured before and after Phonological Components Analysis (PCA) treatment [65], and they were inspected in relation to connectivity in a group of healthy controls (HC). Three core language-related regions in the left hemisphere and their right hemisphere homologues included the following models: the lateral temporal cortex (LTC) as well as the dorsal IFG (dFIG) and ventral IFG (vIFG), which are thought to be preferentially engaged in phonological and semantic processing, respectively [41,42,66,67,68,69]. Within this network architecture, both compensation and normalization patterns may be expected to occur following the phonologically focused therapy. Changes that increase similarity to normal-like connectivity are interpreted as normalization, whereas divergence from the normal-like pattern is interpreted as compensatory reorganization within the language network. While we mostly expect treatment-related changes in the phonological task, which are associated with the phonological treatment [70,71], we also expect to find generalization to semantic processing. Word production models suggest that both semantic and phonological processing levels must be activated to enable word retrieval [72,73]. The reciprocal activation of phonological and semantic representations during naming therapy [74] might explain the fact that both semantic- and phonological-focused therapies have been found to improve naming abilities in many patients with aphasia [70,75,76,77,78,79,80]. Such generalization was also found in our group’s work [10,81]. Our specific hypotheses were as follows:

1. In the phonological task, we mainly expect left intra-hemispheric connectivity in the HC group, as phonological processing is more left lateralized in healthy adults [32,34]. Based on this assumption, we predict that in the phonological task, treatment-related changes among PWA in the left hemisphere will reflect normalization (i.e., will increase resemblance to HC), whereas changes in the RH will reflect compensation (i.e., will differ from HC connectivity);
2. In the semantic linguistic task, however, we expect bilateral connectivity in HC [30,31,32]. Based on this assumption, in the semantic task, bilateral treatment-related changes in PWA are expected to reflect normalization (i.e., increased resemblance to healthy participants);
3. In HC, the semantic task is expected to involve the connections with bilateral ventral IFG, whereas the phonological task is expected to involve the connections with left dorsal IFG, since such functional subdivision of the IFG is evident in healthy individuals [41,42]. Accordingly, among PWA, treatment-related changes are expected to follow this pattern bilaterally, as contralateral right homologous regions were found to be involved in language processing following left hemisphere stroke [13,24,82,83].

## 2. Materials and Methods

This is a re-analysis of previously published data. Approval for the secondary analysis of these data was obtained from the Research Ethics Board at Baycrest Centre, Toronto, Canada. Participants were part of previous studies investigating the efficacy of language treatment [65] and its neural correlates [10,81]. For consistency with the previous studies, we kept the original labels of the individual participants. 

### 2.1. Participants

Four PWA (three males, mean age 67) were included in this study. All had experienced a single left-hemisphere stroke and were at least one-year post-onset at the time of enrollment. No participant presented with a significant apraxia of speech, and none was receiving formal speech-language therapy at the time of testing. Table 1 shows demographic information, diagnosis, and treatment effect size. A group of eight healthy controls (HC) was also included (five males, mean age 58). Two additional participants with aphasia were scanned as part of the original studies as untreated controls but were excluded from this analysis due to a very low explained variance of DCM. All participants were English speakers, right-handed, and had normal or corrected to normal vision. Hearing was within normal limits in at least one ear. Exclusionary criteria included a history of drug or alcohol abuse or a major psychiatric or neurological illness prior to the stroke. All participants provided written informed consent to participate in this investigation.

### 2.2. Anomia Treatment

The four individuals with aphasia received PCA treatment [65] to improve their word-finding abilities. In short, the PCA treatment consists of presenting a target picture in the center of a chart and asking the participant to name it. Subsequently, irrespective of the patient’s ability to name the target, they are asked to provide or choose five phonological components related to the target: a word that rhymes with it, the word’s first sound, another word that starts with the same first sound, the word’s last sound, and the number of syllables. Once this was completed, the patient was asked to name the target again. Then, the examiner reviewed all the phonological components and asked the patient to name the target word a third time. Stimuli were the same as those used in Leonard et al. [65] and consisted of 105 colored photographs of nouns. To establish a baseline, participants were asked to name each item in three consecutive sessions before treatment. Treatment sessions were of approximately one hour in duration and occurred three times a week for approximately 10 weeks. 

### 2.3. Behavioral Results

A complete presentation of the treatment results is available in the original papers [10,81]. To summarize, all treated patients demonstrated significantly improved naming of treated words post-treatment, and these gains were maintained at a follow-up assessment administered four weeks post-treatment. Evidence of generalization to untreated items was significant for P2 [81] and P5 [10]. The results also indicate that there was no change in naming performance in the interval between the two scans for either of the two untreated patients [10]. The effect size for treated items was calculated for all participants by comparing the difference between the post-treatment and baseline means as a function of the baseline standard deviation, following the method of Busk and Serlin [85]. See Table 1. 

### 2.4. fMRI Task

All participants underwent two fMRI scans (S1 and S2), with an average interval of 3.5 months between them. Two tasks, a phonological and a semantic task, were used in the scanner in separate runs of block design, each consisting of a linguistic condition and a perceptual control condition [10]. Briefly, in the semantic task run, the linguistic condition was a semantic judgement task, consisting of 24 trials, with stimuli taken from the Pyramids and Palm Trees Test [86]. In each trial, three pictures were presented: one on top and two on the bottom, and participants were required to indicate, with a keypress, which bottom picture was related in meaning to the top picture. The perceptual control condition of the semantic task consisted of 24 trials, in which three pictures of the same object were presented and oriented as above. One of the bottom pictures was the same size as the top picture, and participants were required to indicate which one was the same size with a keypress. 

In the phonological task run, the linguistic condition was a rhyming judgment task, using 24 pictures from the PALPA 14, Rhyme Judgment Requiring Picture Selection, subtest [87]. In each trial, two pictures were presented side by side, and participants were required to indicate, with a keypress, whether the words rhymed. The perceptual control condition of the phonological task consisted of 24 trials, in which two pictures of the same object were presented side by side. Participants were required to decide whether the two pictures were the same size. In both the phonological and semantic tasks, a low-level baseline condition was also included, in which a fixation cross was presented in the center of the screen, and participants simply pressed a button when it appeared.

### 2.5. fMRI Data Acquisition

Scans were obtained using a 3.0 Tesla system (Signa Eclipse, GE Medical Systems, Milwaukee, WI). A T1-weighted volumetric anatomical MRI was obtained for each participant (124 axial slices, 1.4 mm, FOV = 22 cm). In addition, for each participant, T2*-weighted functional images were acquired using a spiral in/out pulse sequence (26 axial slices, 5 mm, TR = 2000 ms, TE = 30 ms, FOV = 20, 64 × 64 matrix). The blood oxygenation level-dependent (BOLD) effect was used to assess brain activation. The semantic and the phonological tasks were each split into two runs. Therefore, each run contained 12 stimuli of each condition (experimental, control, and baseline), divided into three blocks of four trials each. Each stimulus was presented for 8 s, resulting in 32 s per block and 288 s per run. 

### 2.6. Image Analysis

Data preprocessing was performed using the Statistical Parametric Mapping software (SPM12; Welcome Centre human neuroimaging, London, UK: https://www.fil.ion.ucl.ac.uk/, accessed on 10 September 2021). Preprocessing steps included (1) motion correction through realignment to the mean image; (2) rigid-body registration of the mean functional image to the T1; (3) segmentation, based on co-registered structural images into gray matter, white matter, and cerebrospinal fluid (CSF); (4) normalization of the functional and structural acquisitions to the Montreal Neurological Institute (MNI) standard space; (5) smoothing with a 6 mm FWHM Gaussian kernel to decrease spatial noise.

Statistical analyses with a General Linear Model (GLM) at the first level were done separately for the phonological and semantic tasks. All three conditions—linguistic, perceptual, and fixation—were modeled across the 4 runs (2 runs in each scan, S1 and S2) in a block design, with motion parameters included as covariates of no interest. High-pass temporal filtering with a cutoff period of 128 s was applied to remove low-frequency noise. Task timings were convolved with the canonical hemodynamic response function (HRF). Second-level group analysis was conducted separately for each group (PWA and HC) and for each task (phonological and semantic) across the 4 runs in order to determine a common set of the volumes of interest (VOIs) for each group and task. The t-contrast *linguistic condition minus perceptual control condition across runs* from the first level was taken to the second-level analysis. The peak activation in a one-sample *t*-test at the group level was used to determine the group reference for the location of individual VOIs for effective connectivity analysis.

### 2.7. VOI Extraction

Table 2 presents the coordinates of the group maxima of the six VOIs for the effective connectivity analysis for both groups and tasks: bilateral dIFGs, bilateral vIFGs, and bilateral LTCs, all which were activated across tasks (phonological and semantic) and groups (PWA and HC). The group maxima were chosen based on the group analysis in the contrast *linguistic minus perceptual across runs*, which was thresholded at an uncorrected *p* < 0.01 and resulted in coordinates for healthy controls only in the semantic task. Therefore, the threshold was eventually relaxed to uncorrected *p* < 0.05 in all other cases.

To select these VOIs at the individual level, individual peak activations at the same contrast (from the first-level analysis) were selected and thresholded at uncorrected *p* < 0.05 with a minimum of four active voxels within a 10 mm search radius of each group peak to account for the large anatomical variability in patients [63]. If no activation was found at this threshold, it was relaxed to uncorrected *p* < 0.01, 0.05, and 0.1 (applied in 7, 1, and 4 VOIs, respectively, out of the 144 VOIs identified across individuals and tasks). Anatomical masks, defined by the WFU PickAtlas in SPM12b, were used to ensure that the IFG VOIs were spatially constrained to the IFG, and that LTC was within superior or middle temporal gyri. Subject-specific eigenvalues were extracted with a 6 mm sphere around individual VOIs coordinates, in both tasks, adjusted for effects of interest. 

### 2.8. Effective Connectivity Analysis

Dynamic Causal Modeling (DCM) [87] is a generative model-based Bayesian approach that measures the coupling between neuronal states within a model and context-dependent changes in terms of strength and directionality. The parameters of a DCM are estimated by means of Bayesian hierarchical inversion, using biophysical priors on the hemodynamic parameters and shrinkage priors on the neural parameters to determine how group-level effects constrain parameter estimates on a subject-by-subject basis. When mean-centered, the A matrix reflects the connectivity across all conditions included in the model, and the B matrix represents the modulatory effects exerted by a specific condition through deviation of that condition from the mean. The C matrix represents the driving input to the system. The results include the connection strength and their probabilities. Extrinsic connections reflect the effect that one region has on another region (in Hertz); thus, positive values are interpreted as excitatory, negative values are interpreted as inhibitory, and a value of zero indicates null recruitment of a connection. In contrast, the intrinsic connections are self-inhibitory connections measured in unitless log-scaling parameters that reflect how susceptible a region is to the influence of other regions. Thus, positive values indicate greater self-inhibition and negative values indicate less self-inhibition and, therefore, greater sensitivity to input from other regions. In the current study, we used DCM with Parametric Empirical Bayes (PEB) [88,89,90,91] to examine the effective connectivity changes associated with language treatment for PWA and compare them to the averaged connectivity of HC. 

### 2.9. DCM Specification and Estimation

**Figure 1:**
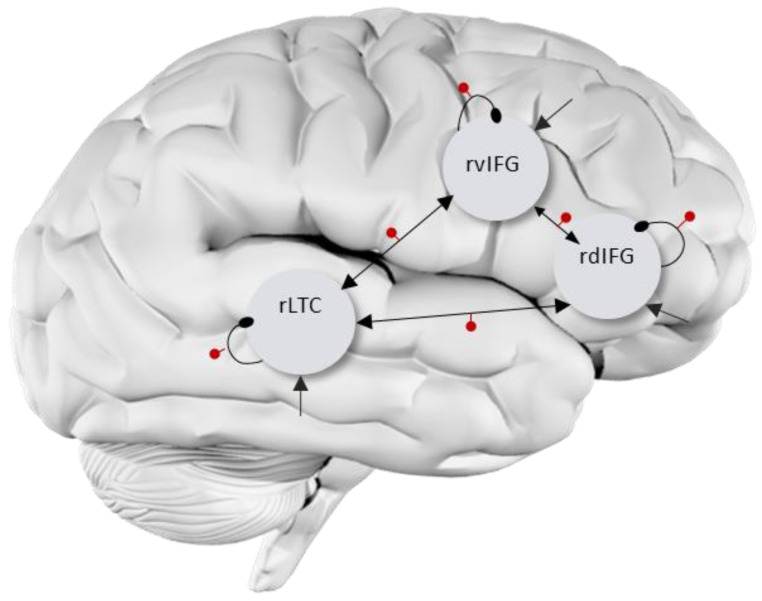
RH DCM full model. Driving inputs (C matrix) are marked by straight arrows into each VOI. Intrinsic self-connections are marked by curved round-end lines. Extrinsic (between-regions) connections are marked by straight arrows between regions. All connections were switched on in both A (all arrows) and B matrices (arrows with red dot) for both phonological and semantic tasks. The bilateral model was similar, with mirror architecture in the left hemisphere, as well as three homotopic connections between hemispheres switched on in A and B matrices.

The semantic and phonological tasks were analyzed separately. The two tasks were identical in structure and timing parameters, so the analysis process is identical. A new SPM model was specified (and not estimated) to serve as the basis for the DCM specification, which included the ‘linguistic condition’ (i.e., phonological or semantic), ‘perceptual-control’ condition (size comparison of object pictures), and an ‘all objects’ condition that included both the linguistic and the perceptual conditions of each task, and it served as the driving input in DCM. In each task, two types of DCM models were specified. (1) The first was a bilateral model, which included six regions: right and left dorsal IFG, right and left ventral IFG, and right and left LTC. All possible intra-hemispheric connections and homotopic inter-hemispheric connections were switched on, meaning they were free to be informed by the data (A matrix). (2) The second was an RH model, which included three regions: rdIFG, rvIFG, and rLTC, with all possible connections free to be informed by the data (see Figure 1). In both models, all the connections between regions and self-connections were free to be modulated by the linguistic task (i.e., the phonological or semantic conditions), resulting in identical A and B matrices. The models were deterministic, bilinear, one-state, without stochastic effects, mean-centered inputs, and time-series fit. For each individual, separate DCMs were specified for each run, resulting in altogether four DCMs per participant in each task (phonological and semantic), two in the pre-treatment scan and two in the post-treatment scan, for each model type (bilateral and RH). We used DCM diagnostics to inspect the percentage of variance (EV) in the data explained by the models, for which a 10% cutoff is typically used [63,89,92]. It showed that for the phonological task in PWA, the bilateral model explained less than 10% of the variance, averaged across participants, while the RH model explained more variance (see Table 3). The semantic task showed low explained variance for both models in PWA. Hence, although our hypotheses included the left hemisphere, because this study focuses on PWA, we chose to continue the analysis only with the RH model, for which the results are more reliable. 

### 2.10. Second- and Third-Level PEB of the RH MODEL

First-level DCMs (four per subject in each task; one for each run) of the RH model were combined into second-level PEB analysis for each individual, separately for each task. These individual PEBs were grouped into a third-level PEB-of-PEBs to capture commonalities and differences among subjects [90]. Bayesian Model Reduction (BMR) was performed to identify parameters that explain unique variance in the fMRI data. Then, Bayesian Model Averaging (BMA), the weighted average of the parameters over models, was used to identify the strength and probabilities of the best reduced model across individuals. The following PEB analyses were conducted separately for the A and B matrices:

#### 2.10.1. PWA Compared to Averaged Connectivity of HC

This analysis was conducted for descriptive purposes. We identified connectivity pre- and post-treatment, as well as treatment-related changes in PWA, and inspected them relative to the average connectivity across time-points in HC. As noted, the two tasks and groups were analyzed separately.

- To explore the average connectivity across the two time-points in each task in HC, as well as treatment-related connectivity changes in PWA, individual second-level PEBs were created, with one covariate for the mean across time-points and one for the difference between time-points. These second-level PEBs were taken to a third-level PEB-of-PEBs with a single covariate representing the mean across the group. The third-level analysis was done separately for PWA, where it was used to determine the change between S1 and S2, and for HC, where it was used to determine the average of S1 and S2.
- To explore connectivity in pre- and post-treatment separately in PWA, another individual second-level PEB was created, with a separate mean-centered covariate for each time-point (S1 and S2). These were taken to a third-level PEB-of-PEBs with a single covariate representing the mean across the group.

These two separate analyses enabled us to inspect which connections changed in PWA following the treatment and whether the change in connectivity from pre- to post-treatment increased or decreased resemblance to the mean connectivity of HC. For connections that changed in PWA but did not survive BMR in the pre- and post-treatment models, we asked whether the direction of the change increased or decreased the resemblance to connection strength in HC.

#### 2.10.2. PWA Compared to Constant Connectivity of HC

Since some of the connections, identified in PWA, may have also changed across time points in HC, the second analysis was meant to determine which of the connections that changed during treatment in PWA remained stable in HC. For this purpose, individual PEBs of both groups from analysis 2.10.1 were directly compared between groups in the third-level PEB-of-PEBs analysis across both groups. We identified connections that survived the interaction between Group X time-point and also showed changes in connectivity across time-points only in the PWA but not in HC. 

## 3. Results

As indicated above, due to the low explained variance by the bilateral model, all the results are reported only for the RH model. All effects that survived BMR are reported in the figures, with those with probability of >0.9 after BMR marked in bold and an asterisk.

### 3.1. Connectivity of HC 

**Figure 2:**
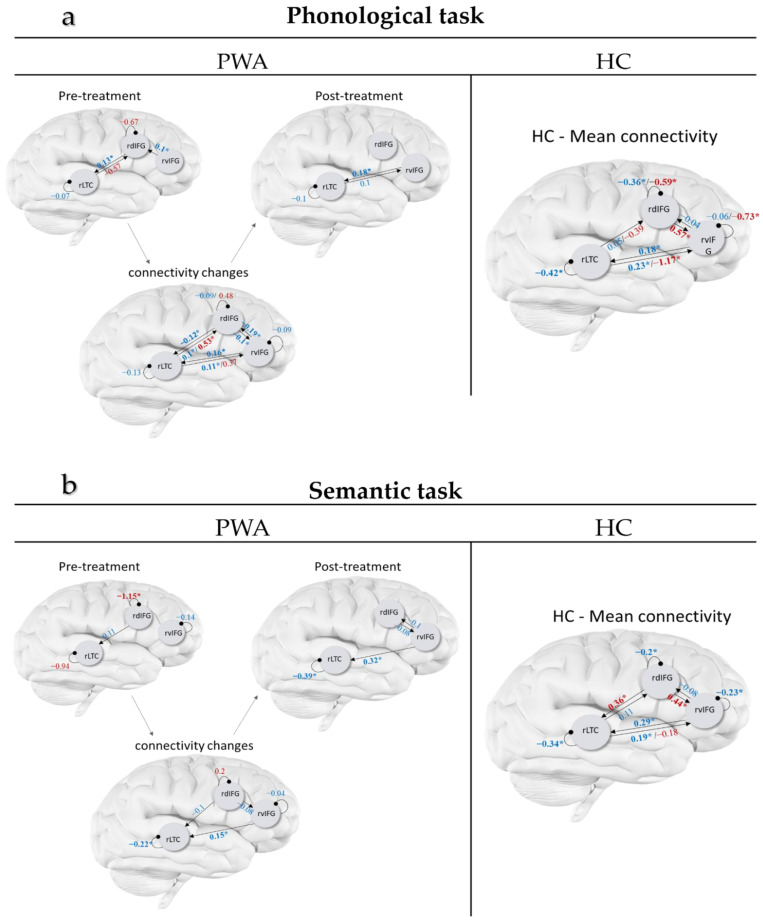
Connectivity after BMR in people with aphasia (PWA; pre-treatment, post-treatment, and changes from pre to post-treatment) and connectivity in healthy controls (HC; averaged across the two scans). Results are shown for the phonological (**a**) and semantic tasks (**b**). Black arrows represent connection direction. Numeric values represent connections strength (in Hz for extrinsic connection and in unitless log-scaling for intrinsic connection), or the change in strength from pre- to post: blue values for averaged connectivity (A matrix) and red for the modulatory effect of the linguistic condition (B matrix). Bolded starred values (*) are connections with probability > 0.9.

Among HC, a similar connectivity architecture survived BMR for both the phonological and the semantic tasks in the A matrix (mean connectivity across linguistic and perceptual control blocks). Specifically, the average connectivity across all conditions and across the two time-points was similar for the phonological and semantic tasks, including all three self-connections (for rdIFG, rvIFG and rLTC) as well as four extrinsic connections: reciprocal excitatory connections between rvIFG ↔ rLTC, an excitatory connection from rLTC → rdIFG, and an inhibitory connection from rvIFG → rdIFG. The strength and probabilities of these connections are depicted in Figure 2a,b (right, values in blue). 

The modulatory effects of the linguistic conditions (i.e., the B matrices) showed some similarities across the phonological and semantic tasks, i.e., both phonological and semantic linguistic conditions increased the connection rdIFG → rvIFG and decreased the connection rLTC → rvIFG beyond the A matrix. There were also some differences; specifically, the phonological condition decreased the self-inhibition in the two frontal areas (rdIFG and rvIFG) and made them more sensitive to afferent inputs, and it decreased the connections from rLTC → rdIFG. In contrast, the semantic judgment condition increased the reciprocal connection rdIFG → rLTC (Figure 2a,b, values in red).

### 3.2. Connectivity of PWA 

To track treatment-related changes in PWA in relation to HC, we inspected whether changes in connections from pre- to post-treatment in PWA, that survived BMR, increased or decreased in resemblance to the mean connectivity across scans in HC. In the phonological task, the connectivity changes across all trials with object stimuli (A matrix) revealed that all three intrinsic and six extrinsic connections changed in PWA following the language treatment (see Figure 2a left, values in blue). Eight of the connections increased in their resemblance to HC connectivity: rdIFG ↔ rLTC, rLTC ↔ rvIFG, rvIFG → rdIFG, and all self-connections. The analyses on the modulatory effects of the linguistic phonological condition (B matrix) showed that the modulatory effect on three connections changed from pre- to post-treatment in PWA: rLTC → rdIFG, rLTC → rvIFG and the self-connection of rdIFG (see Figure 2a left, values in red), and none of them increased their resemblance to HC.

In the semantic task, the connectivity across all trials with object stimuli (A matrix) changed following treatment in PWA in two intrinsic and three extrinsic connections (see Figure 2b left, values in blue). Of these, four increased in resemblance to HC: rdIFG → rLTC, rvIFG → rLTC, and self-connections of rLTC and rvIFG. The modulation of the linguistic semantic condition (B matrix) changed during treatment in PWA only for the self-connection of rdIFG (see Figure 2b left, values in red). This effect increased in its resemblance to HC. 

**Figure 3:**
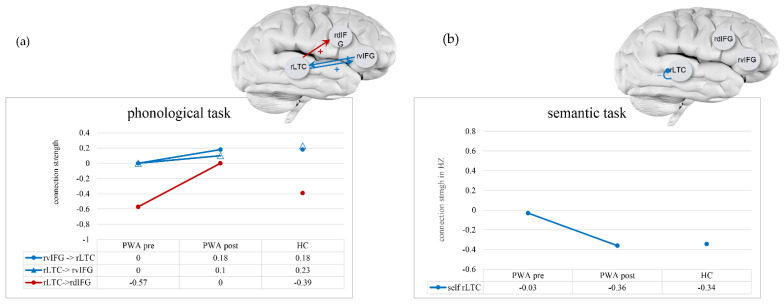
Connections that changed during treatment in patients with aphasia (PWA) and remained stable in healthy controls (HC) in the phonological (**a**) and semantic (**b**) tasks. Connection strength (in Hz for extrinsic connection and in unitless log-scaling for intrinsic connection) are depicted on the Y-axis and in the table, for pre- and post-treatment for PWA and as the average across time points in HC. Blue indicates the average across all trials with objects (A matrix), and red indicates the modulation of the phonological or semantic conditions (B matrix). A plus sign (+) indicates increased excitation; a minus sign (−) in the self-connection indicates decreased inhibition.

In the second analysis, we asked which of the above changes from S1 to S2 in PWA were exclusive to PWA, which would be reflected in the fact that the connections remained stable across the two time-points in HC. The analysis of the averaged connectivity across all trials with objects (A matrix) in the phonological task showed that two out of five connections met this criterion: bidirectional rvIFG ↔ rLTC. These two connections increased in excitation following treatment, and thus, as indicated above, they increased in their resemblance to HC (see Figure 3a, blue lines). The analysis of the modulatory effects exerted by the phonological linguistic condition (B matrix) showed that only the modulation of one connection, Rltc → rdIFG, changed exclusively in PWA. It had strengthened following treatment but did not increase in its resemblance to HC (see Figure 3a red line). In the semantic task in the A matrix, only one connection changed exclusively in PWA following treatment: the self-connection for rLTC became less inhibitory, thus, increasing its resemblance to HC (see Figure 3b, blue line). No modulatory effect in the linguistic semantic condition changed exclusively in PWA. 

## 4. Discussion

The main goal of the current study was to investigate treatment changes in effective connectivity among patients with chronic aphasia measured during performance on phonological and semantic tasks. Of the two DCM models considered, the examination of the bilateral model was discontinued due to low explained variance for PWA, rendering potential results less reliable. As a result, we focused the analyses on connectivity changes within the right hemisphere model, including dorsal IFG (rdIFG), ventral IFG (rvIFG), and lateral temporal cortex (rLTC). We predicted that the effect of a phonological treatment will be evident not only in the network associated with phonological processing but will also generalize to semantic processing. Furthermore, in the phonological task, changes in the right hemisphere for PWA were expected to differ from HC and to involve rdIFG. However, in the semantic task, changes in connectivity in the RH of PWA were expected to resemble those of HC and involve right ventral IFG. 

### 4.1. Connectivity of HC Participants

The results of the analyses revealed that among the HC group, the connectivity across perceptual and linguistic conditions (A matrix), averaged across the two time-points, resulted in similar architecture in the semantic and phonological tasks: excitatory bidirectional connection rLTC ↔ rvIFG, along with all regions being sensitive to afferent inputs, as reflected in self-connections. This architecture reflects the common processing demands across all four conditions, which included looking at pictures of real objects. Despite differences between the requirements of the linguistic and perceptual conditions, all of them may have resulted in silent automatic retrieval of the object names [93,94,95], which may explain the recruitment of RH homologs of language regions. Although typically strongly left-lateralized, a bilateral network has been found to be involved in object naming in healthy individuals [96,97].

We also examined the modulatory effects of the linguistic conditions (B matrices), which are connections that were selectively enhanced or inhibited by the phonological rhyming or semantic related judgment conditions, as compared to the average connectivity across the perceptual and linguistic conditions (A matrix) in each task. This analysis showed both shared and unique effects in the two tasks. The decreased excitatory connection from the temporal to the dorsal IFG in the phonological condition, and the increased excitatory connection from rdIFG to the temporal region in the semantic condition are consistent with the notion that the right hemisphere is involved in semantic rather than phonological processing in healthy individuals. 

Nevertheless, we also found an increased excitatory connection between the two frontal regions (rdIFG → rvIFG) in both linguistic conditions and reduced self-inhibition in both frontal areas in the phonological condition. These effects are not consistent with the above hypothesis and suggest greater involvement of the right frontal regions in phonological processing than previously suspected. Alternatively, the modulation of connections in the RH by the phonological and semantic conditions may reflect the involvement of domain-general control processes in these linguistic decisions [33,98]. This notion is supported in part by greater functional connectivity in the RH among healthy elderly individuals, suggesting they rely more on domain-general control processes during linguistic processing compared to younger adults [99]. In their meta-analysis, Hartwigsen et al. [100] demonstrated a functional posterior–anterior axis in the right IFG for non-linguistic tasks, with posterior parts associated with motor and perception-related functions, while the anterior parts are involved in abstract cognitive functions. Thus, the increase in the rdIFG → rvIFG connection by both phonological and semantic conditions in the current study may reflect domain-general processes associated with the formation of abstract representations that support linguistic processing. 

### 4.2. Connectivity of PWA

With respect to the participants with aphasia, overall, their connectivity pattern demonstrated clear support for the right hemisphere model compared to the bilateral model across the pre- and post-treatment scans. This is not surprising, given that the lesion in the LH disrupts both intra- and inter-hemispheric connectivity in the LH. The activation of right homolog structures of classic language regions has been described repeatedly in neuroimaging studies of patients with left hemisphere lesions [13,24,82,83]. This finding is not consistent with a previous effective connectivity study [101], wherein PWA showed preference for an LH over a bilateral structured model. Nevertheless, the above study did not examine a RH-only model. Overall, our results from the RH model show that connectivity during the phonological task showed more changes from pre- to post-treatment, compared to the semantic task. This may be due to the phonological focus of the PCA treatment. Below, we will discuss connectivity changes in PWA following treatment in relation to the HC group. 

In both the phonological and semantic tasks, changes in the connectivity across perceptual and linguistic conditions (A matrix), following treatment, occurred in multiple connections and mostly resulted in increased resemblance to that of healthy controls. More specifically, the connection changes from pre- to post-treatment that were evident in PWA while being stable in HC are an increase in the excitatory reciprocal connection between rLTC ↔ rvIFG in the phonological task and the decrease in self-inhibition of rLTC in the semantic task, both of which reflect normalization. These treatment-related changes, which are not specific to the linguistic condition, may be related to enhanced silent automatic retrieval of object names, which may occur also in perceptual trials [93,94] and may be supported by the naming treatment. 

We also found treatment-related changes in the modulatory effect of the phonological condition (B matrix) beyond the average connectivity of all object trials (A matrix). The modulation of the phonological rhyming condition on the connection from rLTC to rdIFG increased in excitation following the treatment in PWA, but it was stable in HC. This change decreased the resemblance between PWA and HC, who showed negative connectivity between these regions during the rhyming task, suggesting that the increase in the modulation of the phonological condition on this connection may have a compensatory role. Given that all the patients improved in naming following the phonological treatment, this increase in connectivity may reflect effective compensation through the recruitment of an atypical connection in the right hemisphere to support phonological processing. This finding is consistent with our hypothesis (#1) and suggests that the connection rLTC to rdIFG, which is not involved in phonological processing in HC, is recruited for this function in patients following treatment. This finding also provides partial support for our hypothesis (#3), suggesting that the dorsal part of right IFG would support phonological processing in PWA, mirroring the role of its LH homologue in phonological processing in healthy individuals [41,42]. An alternative interpretation of the above finding is based on the possibility that the RH involvement in phonological processing in the HC group reflects domain-general control processes [33,98]. According to this interpretation, treatment-related changes in patients, in the phonological condition in the RH, also reflect changes in domain-general control processes, which support performance on the rhyming task in the scanner. Such processes may have been enhanced by the meta-linguistic aspects of the PCA treatment. However, this interpretation is less likely given the specificity of this effect to the phonological condition, and the absence of such change in the semantic condition. In a recent study [102], direct and indirect structural connections between the left IFG opercularis and left middle temporal gyrus (MTG) were associated with gains following semantic naming treatment in participants with aphasia. While our study could not test changes to this connection in the LH, it suggests that changes to the right homologue of this connection also play a role in language recovery. 

In the semantic judgement condition (B matrix), there were no treatment-related changes that survived BMR and that were stable in HC across time-points. This is in contrast to our hypothesis (#2) that changes related to the phonological treatment would generalize to semantic processing. Nevertheless, the absence of specific changes in the semantic task may also be related to the low explained variance by the DCM models for this task, making all the findings in the semantic task less reliable. 

### 4.3. Limitations

There are several limitations to consider in the current study. First, there are well-known challenges in the recruitment of PWA for treatment and imaging studies. Nevertheless, the small sample size of PWA in this study limits the generalization of its findings. Second, the selection of the RH model over the bilateral model in both groups was done because of the focus on the PWA and in order to enable comparability between groups. Nevertheless, while the RH model explained on average more than 10% of the variance even in HC, the bilateral model, which showed greater fit in HC, may have resulted in different connectivity patterns. Finally, although the ROIs were selected based on voxels showing above threshold activation in each of the linguistic tasks, the variance explained by the DCM models in the semantic task was very low. As suggested above, the low explained variance in the semantic task may explain the fewer and weaker modulatory effects and lack of treatment-related changes in this task. 

## 5. Conclusions

In the current study, we explored changes in effective connectivity in RH language homologue regions among PWA, following phonological treatment, compared to a group of HC. Connectivity patterns in HC suggest recruitment of the RH network for these phonological and semantic tasks, which may reflect either linguistic or domain-general control processes supporting lexical judgments. Treatment-related changes in PWA in the averaged connectivity across conditions in both tasks showed increased resemblance to HC, suggesting that the phonological naming treatment normalized the network associated with automatic silent retrieval of object names. In the phonological linguistic condition (rhyming task), treatment-related changes decreased in resemblance to HC, indicating compensatory reorganization in RH connectivity. While we did not find evidence of generalization of the phonological treatment effects to brain connectivity during semantic processing, this may be due to methodological limitations in the study. Sarasso et al. [58] found that the treatment-related changes in structural connectivity of PWA tend to increase resemblance to the normal connectivity network. Our findings from effective connectivity suggest that reorganization is a complex process. In contrast to the dichotomous view of normalization in the left hemisphere versus compensation in the right hemisphere, our work supports an active and dynamic role of right hemisphere connectivity in chronic aphasia, which is engaged in language recovery. Although the right hemisphere may not be unitary in its involvement in language processing post-stroke, the current data suggest that both normalization, evidenced by increased resemblance to HC, and compensation, evidenced by the emergence of atypical connectivity, seem to play a simultaneous role in the neural reorganization of the language network among elderly people with chronic aphasia. Further work of brain effective connectivity changes during language rehabilitation is required in order to increase our understanding of these reorganization processes.

## Figures and Tables

**Table 1:** Participant characteristics and scores (percent correct) on background tests for patients with aphasia.

	P2 ^a^	P4 ^a^	P5 (ATr1 ^a^)	P6 (Atr2 ^a^)
Age (years)	81	64	50	73
Education (years)	14	12	16	12
Gender	M	M	F	M
Time post onset (years)	1	1.75	3.5	4
Lesion site	LMCA	Left frontotemporal	Left posterior frontal, temporal and parietal	Left frontotemporal
Aphasia type ^b^	Broca’s	Broca’s	Broca’s	Mixed non-fluent
BNT [mean 94, normal range 78.3–100] ^c^	17	13	13	40
Mean effect size across treated items	3.94	1.53	3.00	3.47

^a^ P2 and P4 correspond to participants 2,4 in Leonard et al. [81]. P5 and P6 [65] were labeled Atr1 and Atr2, respectively, in Rochon et al. [10]. ^b^ Aphasia type was determined based on Boston Diagnostic Aphasia Examination (BDAE; [84]. ^c^ Boston Naming Test 60-item version.

**Table 2:** Group coordinates used as reference for VOI selection in each group and task.

	**Phonological Task**								
	Participants with aphasia					Healthy Controls			
	X	Y	Z	BA		X	Y	Z	BA
rvIFG	36	30	2	45		50	20	−8	47
lvIFG	−36	28	6	45		−42	20	−4	47
rdIFG	50	18	32	44		38	22	30	9
ldIFG	−56	22	28	44		−48	32	24	9
rLTC	56	−26	4	22		64	−30	−4	21
lLTC	−68	−40	8	21		−64	−40	8	21
	**Semantic task**								
	Participants with aphasia					Healthy Controls			
	X	Y	Z	BA		X	Y	Z	BA
rvIFG	38	38	−6	47	52	20	2	45	38
lvIFG	−46	26	−2;	45	−36	30	−8	47	−46
rdIFG	58	28	16	9	38	16	22	44	58
ldIFG	−54	34	16	46	−42	14	28	44	−54
rLTC	52	−8	0	41	64	−46	6	21	52
lLTC	−62	−26	−2	21	−66	−42	6	21	−62

Legend: r—right, l—left, v—ventral, d—dorsal, IFG—inferior frontal gyrus, LTC—lateral temporal cortex.

**Table 3:** Percent of explained variance by the DCM. The average [and range] per run across participants are presented.

Task	Group	RH Model	Bilateral Model
Phonological task	PWA	14.49 (6.0–21.3)	8.22 (6.2–11.7)
	HC	12.10 (5.73–20.1)	16.98 (9.6–30.7)
Semantic task	PWA	4.49 (2.2–6.8)	5.1 (2.8–7.8)
	HC	9.82 (5.3–21.7)	16.66 (9.5–22.4)

## Data Availability

The data presented in this study are available on request from the corresponding author. The data are not publicly available due to ethical reasons.
